# Widespread Forest Vertebrate Extinctions Induced by a Mega Hydroelectric Dam in Lowland Amazonia

**DOI:** 10.1371/journal.pone.0129818

**Published:** 2015-07-01

**Authors:** Maíra Benchimol, Carlos A. Peres

**Affiliations:** 1 School of Environmental Sciences, University of East Anglia, Norwich Research Park, Norwich, United Kingdom; 2 PPG Ecologia e Conservação da Biodiversidade, Laboratório de Ecologia Aplicada à Conservação, Universidade Estadual de Santa Cruz, Rodovia Jorge Amado km 16, Ilhéus, BA, Brazil; 3 Capes Foundation, Ministry of Education of Brazil, Caixa Postal 250, Brasília, DF–Brazil; University of Delhi, INDIA

## Abstract

Mega hydropower projects in tropical forests pose a major emergent threat to terrestrial and freshwater biodiversity worldwide. Despite the unprecedented number of existing, under-construction and planned hydroelectric dams in lowland tropical forests, long-term effects on biodiversity have yet to be evaluated. We examine how medium and large-bodied assemblages of terrestrial and arboreal vertebrates (including 35 mammal, bird and tortoise species) responded to the drastic 26-year post-isolation history of archipelagic alteration in landscape structure and habitat quality in a major hydroelectric reservoir of Central Amazonia. The Balbina Hydroelectric Dam inundated 3,129 km^2^ of primary forests, simultaneously isolating 3,546 land-bridge islands. We conducted intensive biodiversity surveys at 37 of those islands and three adjacent continuous forests using a combination of four survey techniques, and detected strong forest habitat area effects in explaining patterns of vertebrate extinction. Beyond clear area effects, edge-mediated surface fire disturbance was the most important additional driver of species loss, particularly in islands smaller than 10 ha. Based on species-area models, we predict that only 0.7% of all islands now harbor a species-rich vertebrate assemblage consisting of ≥80% of all species. We highlight the colossal erosion in vertebrate diversity driven by a man-made dam and show that the biodiversity impacts of mega dams in lowland tropical forest regions have been severely overlooked. The geopolitical strategy to deploy many more large hydropower infrastructure projects in regions like lowland Amazonia should be urgently reassessed, and we strongly advise that long-term biodiversity impacts should be explicitly included in pre-approval environmental impact assessments.

## Introduction

Hydroelectric dams are rapidly emerging as the new villain in the myriad of anthropogenic threats to tropical forest biotas. Dams displace indigenous communities [1], disrupt the natural flow of rivers [2], critically affect fish populations [3], release vast amounts of greenhouse gases [4], and promote wholesale deforestation and fragmentation of pristine forests [5]. From China to Brazil, hydroelectric dams have been built at an unprecedented scale to supply burgeoning energy demands [6]. More than 945,000 dams higher than 15 m have been built worldwide, altering >50% of all major rivers [7]. In South America alone, some 2,215 new hydroelectric dams are expected to be erected within the next few years [8]. Assessing the true impacts of hydropower infrastructure on natural ecosystems has therefore become an urgent priority for the environmental policy agenda of emergent economies.

In Brazilian Amazonia, over 10 million ha of forests are expected to become permanently inundated following the planned construction of new dams [9], potentially leading to a colossal impact on both terrestrial and aquatic biotas at regional scales. Hydroelectric dams in lowland forests typically resort to low-declivity river basins, submerging vast upstream areas per unit of megawatt output generated, thereby often creating vast archipelagos of forest isolates. Land-bridge islands formed by these artificial lakes may experience stronger isolation effects than forest remnants embedded within a terrestrial landscape, largely because the open-water matrix is invariably less permeable to terrestrial organisms than pastures and second-growth vegetation (e.g. [10–12]). However, despite an embryonic set of studies investigating the long-term impacts of major dams on biodiversity worldwide [13–15], the extinction dynamics of archipelagic landscapes created by hydroelectric reservoirs remains poorly understood in tropical forest regions, specially for Amazonian reservoirs (but see [16–17]).

Terrestrial vertebrates are pivotal components of tropical forest dynamics through their ecological roles as hyper-consumers, large predators, frugivores, seed dispersers, and structural habitat modification [18–19]. They are also widely hailed as pinnacle conservation icons, contributing much public charisma for tropical forest conservation. In Amazonia, hunting pressure is the strongest driver of local extinctions of medium and large mammals stranded in fragmented landscapes [20–21]. With the exception of primates [16], no study has assessed the long-term impacts of a hydroelectric reservoir on medium and large-bodied vertebrates at the community level in Amazonian land-bridge islands. Yet this is required to both elucidate the positive or negative effects of existing dams on biodiversity, and refine environmental impact assessments of future dams.

Here, we provide the first quantitative assessment of how medium- and large-bodied arboreal and terrestrial vertebrate assemblages (including 35 mammal, bird and testudine reptile species) responded to the drastic 26-year post-isolation history of alteration in landscape and habitat quality by a mega hydroelectric dam in Central Brazilian Amazonia. The notorious Balbina Hydroelectric Reservoir (BHR) inundated 312,900 ha of primary forests, subsequently converting all emerged areas into an archipelago of 3,546 islands. Using a combination of four complementary sampling techniques, we conducted quantitative faunal surveys at 37 pre-selected islands (size range = 0.83 ─ 1690 ha) and three mainland continuous forest sites to examine how patterns of species persistence are related to seven habitat quality, forest patch and landscape metrics. We document the extent of local vertebrate extinctions within islands, build a model to predict extinction rates across all unsurveyed islands, and identify priority areas for vertebrate conservation within the reservoir. This study serves a critical policy role at a time of greatly augmented investments in hydropower development in Amazonia in informing the scientific community and the wider public about the detrimental impacts of major dams on forest biodiversity.

## Materials and Methods

### Study sites

Following the completion of the Balbina Hydroelectric Dam in October 1986, a reservoir area of 443,700 ha was formed, comprising 3,546 variable-sized land-bridge islands ranging from 0.194 to 4878.1 ha. To offset the forest habitat loss, the reservoir and adjacent mainland continuous forests were protected from 1990 with the creation of the Reserva Biológica (REBIO) do Uatumã, the largest Biological Reserve in Brazil. With nearly 940,000 ha, the REBIO protects a vast continuous forest area and all islands on the left bank of the Uatumã river. Islands on the right bank are under permanent preservation but are not strictly protected, allowing recreational fishing. Due to the homogeneous habitat matrix and isolation time, major hydroelectric lakes are excellent island biogeography experimental landscapes with many land masses isolated simultaneously [15, 22–23]. The Balbina Hydroeletric Reservoir (BHR) has several advantages compared to other archipelagic and terrestrial fragmented landscapes, including a long-term relaxation time, a large number of habitat patch replicates containing a wide range of patch sizes (< 1 ha to 4,000 ha), and effective protection from anthropogenic disturbance, including logging and hunting [16]. None of the surveyed islands had experienced a recent history of hunting pressure. This is based on information provided by the reserve surveillance team and the conspicuous lack of evidence of hunting in these islands over the last 8 years (the period of time our field team has been working at the BHR). However, ephemeral understorey fires accidentally affected much of the BHR landscape during the severe El Niño drought of December 1997 to January 1998. A single source of ignition, attributed to a local fisherman, rapidly propagated into previously unburnt primary forests and islands across parts of the reservoir.

We used two cloudless georeferenced 30-m resolution Landsat ETM+ images (230/061 and 231/061; year 2009) to carefully pre-select 37 forest islands, ranging in size from 0.83 to 1690 ha, to be surveyed on the basis of their size, degree of isolation and spatial distribution within the reservoir, representing a wide range of BHR island configurations [24]. We also selected three widely distributed ‘pseudo-control’ continuous forest sites (CFs) in the adjacent mainland area (S1 Fig). Surveyed islands and mainland sites, which spanned a study area of ~396,400 ha, were spaced by at least 1 km from one another to maximize spatial independence.

Sampling was carried out under permit No. 12344–1 issued by the Instituto Chico Mendes de Conservação da Biodiversidade (ICMBio/MMA).

### Vertebrate surveys

Between June 2011 and December 2012, we surveyed the entire midsized to large diurnal and nocturnal vertebrate fauna that is amenable to at least one of four field sampling techniques (line-transect censuses, indirect sign surveys, armadillo surveys and camera trapping). We first listed all terrestrial and arboreal vertebrate species >100g expected to occur in the entire study landscape, based on field guides (e.g. [25–26]), IUCN range polygons [27] and our own extensive personal knowledge, including previous studies at Balbina. These included primate, carnivore, xenarthran, ungulate and rodent mammal species, plus four large terrestrial bird and two tortoise species (S1 Table). One to five variable-length transects were cut within each island, according to their size and shape so that a representative island area could be covered [24]. On each continuous forest (CF), we established three parallel 4-km linear transects, separated from each other by 1 km (S1 Fig).

Line-transect surveys consisted of quiet walks conducted by two previously trained observers at a constant speed (~1.0 km/h) following a standardized protocol [28]. Surveys were carried out in the morning (06:15–10:30) and afternoon (14:00–17:30), and were discontinued during rainy periods. We conducted four line-transect surveys on each sampling site during each year of study (2011 and 2012), separated by intervals of at least 30 days, minimizing possible effects of time of day and seasonality. On return walks, we also conducted surveys of all signs of vertebrate activity (tracks, digging, feces, hair, burrows and partly consumed fruits). These were searched along the transect, and the species identification recorded. Whenever signs of the same species were encountered, we considered a minimum distance of 500m for signs to be defined as spatially independent. Armadillo burrows deeper than 50cm encountered within a distance of 5m either side of each transect were searched and recorded only once per year, and measured following [29]. We used Reconyx HC 500 Hyperfire digital camera traps (CTs) to complement our vertebrate surveys. All CT stations at each forest site were sampled over a 30-day period during each year of study (2011 and 2012). We deployed two to ten CTs at each island (mean [SD] = 4.38 [3.21], according to island size, and 15 CTs at each CF site, with five on each transect [24]. CTs were unbaited, spaced by at least 500 m (except for small islands), and placed 30–40 cm above ground. We configured all CTs to obtain a sequence of five photos for each animal recorded, using 15-sec intervals between records. However, we only considered records of the same species as independent if intervals between photos exceeded 30 min, or if different individuals could be recognised on the basis of natural marks. Observational sampling was never conducted at any forest site during periods of camera-trapping to minimise site disturbance.

In total, 81 transects of lengths ranging from 0.5 to 4.0 km (mean [SD] = 2.71 km [3.32], total = 108.5 km) were implemented. These were each surveyed twice (2011 and 2012) over a total effort of 1,168 km walked during line-transect censuses; 1,168 km of sign surveys; and 217 km of armadillo-burrow counts. We obtained a total of 12,420 CT-days (mean [SD] = 310.5 [251.83], range = 120–900 CT days/site) from 207 camera-trapping stations [24].

### Forest metrics

We adopted a patch-landscape approach [30], surveying focal patches but including variables at both patch and landscape scales in the analysis. We used high-resolution multi-spectral RapidEye imagery (5-m resolution with 5-band colour imagery) to extract patch and landscape variables. RapidEye consists of a constellation of five identical satellites producing 5-m resolution with 5-band colour imagery. We selected tile images on the basis of low cloud cover (<10%) and months matching our field sampling. A total of 28 different tiles from March 2011 to September 2012 were used, covering an area of 698,000 ha. Using ArcMap (version 10.1), we conducted a semi-supervised classification to obtain four land cover classes (closed-canopy forest, open-canopy forest, bare ground, and water). At the patch scale, we calculated island area, total forest area (excluding bare ground), closed-canopy forest area, nearest distance to a continuous forest site, and island shape (perimeter: area ratio; [24]). These patch metrics were obtained for all 3,546 BHR islands, including both surveyed and unsurveyed islands. At the landscape scale, we considered multiple buffers (250m, 500m and 1000m) outside the perimeter of each island and mainland forest sites, and quantified the percentage of both total forest cover and closed-canopy forest within the buffer, and modified the McGarigal et al. [31] proximity index by considering the total size of any land mass within the buffer, rather than excluding land areas outside the buffer within patches encompassed by the buffer. Within each forest site, we calculated the percentage of closed-canopy forest, a measure of burn severity (defined as a composite ordinal score based on the extent to which each forest site was affected by surface fires, and the number of charred trees and char height marks on each tree), and the aggregate basal area of all trees ≥10 cm dbh [diameter at breast height] bearing fleshy fruits based on 87 quarter-hectare permanent forest plots inventoried at all forest sites [17, 24].

### Data analysis

We analysed all occupancy data in terms of species presence/absence (P/A). Combining all four sampling techniques at the 40 surveyed sites, we recorded a total of 35 midsized and large vertebrate species (S1 Table) on the basis of 5,765 visual and acoustic records during line-transect censuses (mean [SD] = 155.8 [219.8], range = 0─1051); 1,850 sign records (mean [SD] = 50.0 [61.9], range = 0─251); 427 armadillo burrows (mean [SD] = 14.72 [15.23], range = 0─47); and 10,110 independent camera trapping photos (mean [SD] = 273.24 [264.6], range = 0─857). Combining all four sampling methods, we then constructed three P/A matrices including all 35 species recorded, initially considering all 40 forest sites, and then disaggregating the data at the scales of either 217 transect segments of 500m (in the case of line-transect censuses, sign surveys, and armadillo surveys) or the 207 camera trapping stations sampled.

We defined a species as ‘present’—at the scales of site, transect segment, or CT station—if it appeared at least once during any of eight census repeats for both line-transect and sign surveys, or during either one of the two annual 30-day CT sessions per CT station. We first estimated the total number of species (species richness) from the sum of all species recorded at each site regardless of sampling technique. To account for potential sampling biases due to unavoidable between-site differences in sampling effort as a function of forest patch size, we also calculated the summed mean (±SD) number of species per sampling technique, defined as the sum of the mean number of species, each of which assigned exclusively to its ‘best’ technique. For this, we first compared the species-specific proportions of occupied sites per technique. We then summed all means and standard deviations (SD) provided by each technique per forest site to obtain an aggregate mean (± SD) species richness per forest site considering all four sampling techniques. We then used a random resampling approach to examine patterns of species richness on the basis of a standardized survey effort across all sites per 500-m transect segment or individual CT station. This was based on a jacknife procedure that resampled either census walk segments or CT stations at each of the 40 forest sites based on 1000 iterations. Finally, we examined species-area relationships (SARs) using both the total number of species and the resampled mean (±SD) species richness against (log_10_ x) forest patch area using both semi-log models and power models, as they perform well in explaining species-area relationships [32–33].

Additionally, we performed nonmetric multidimensional scaling (NMDS) ordinations based on the Bray-Curtis similarity matrix of species composition using the combined occupancy data based on all four sampling techniques. We also obtained a measure of aggregate biomass for each forest site, by summing the estimated body mass of all species occurring at each site based on compiled data [34] and an indigenous hunting study conducted ~80 km from the BHR where most game carcasses were weighed [35]. We also used four species-specific attributes (body mass, trophic status, locomotion mode and group size) to quantify the vertebrate functional diversity (FD) of each forest site based on a dendrogram approach [36]. This method encompasses four steps: (1) a design of the trait matrix; (2) a conversion of this matrix into a distance matrix; (3) a hierarchical clustering of the distance matrix to produce a functional dendrogram; and (4) a calculation of the total branch length of the dendrogram, providing a continuous FD metric. We used the Euclidean distance and the unweighted paired-group clustering method using arithmetic averages, and performed the analysis using Petchey’s [37] R code. We then investigated patterns of species composition, biomass and FD in relation to forest patch area through semi-log regression models, considering the NMDS measure of species composition, aggregate biomass, and FD as response variables.

We further performed Generalized Linear Models (GLMs) to examine the vertebrate species richness to the explanatory variables. We first performed a Pearson’s correlation analysis between patch, landscape, and habitat quality variables across all 37 islands and three mainland sites, and then excluding the latter, retaining weakly correlated variables (r ≤ 0.70). Because some variables were highly related, we performed stepwise linear regression models to select the best patch- and landscape-scale predictors to be included in further analyses ─ log_10_ forest area (hereafter, ‘area’), patch shape, distance to continuous forest site (‘isolation’), proximity index within 500-m buffers (‘proximity’); percentage of closed-canopy forest within the patch (cc%), burn severity (‘burn’), and basal area of trees bearing fleshy fruits (‘ba_*ff*_’). We then tested for multicollinearity among these variables by deleting the least moderately redundant or collinear Variation Inflation Factors (VIF ≥ 5; [38]) We performed species richness GLMs considering: (1) all 40 forest sites, including fixed effects available for CFs (area, burn, cc% and ba_*ff*_); (2) the 37 islands only, with all fixed effects retained (area, isolation, shape, proximity, burn, cc% and ba_*ff*_); and (3) only 15 islands smaller than 10 ha, retaining all fixed effects but excluding forest area. We ran all predictor subsets using the ‘*MuMIn*’ package [39] and obtained model-averaged estimates. We further determined both the relative importance of each variable using hierarchical partitioning (HP) and unique fractions of variation explained for each significant variable using variance partitioning (VP).

Finally, we used empirical models based on key patch and landscape variables that best explained patterns of species richness at surveyed islands to predict local extinction rates for all forest vertebrate species across the entire BHR landscape.

## Results

### Determinants of species richness and diversity

**Fig 1:**
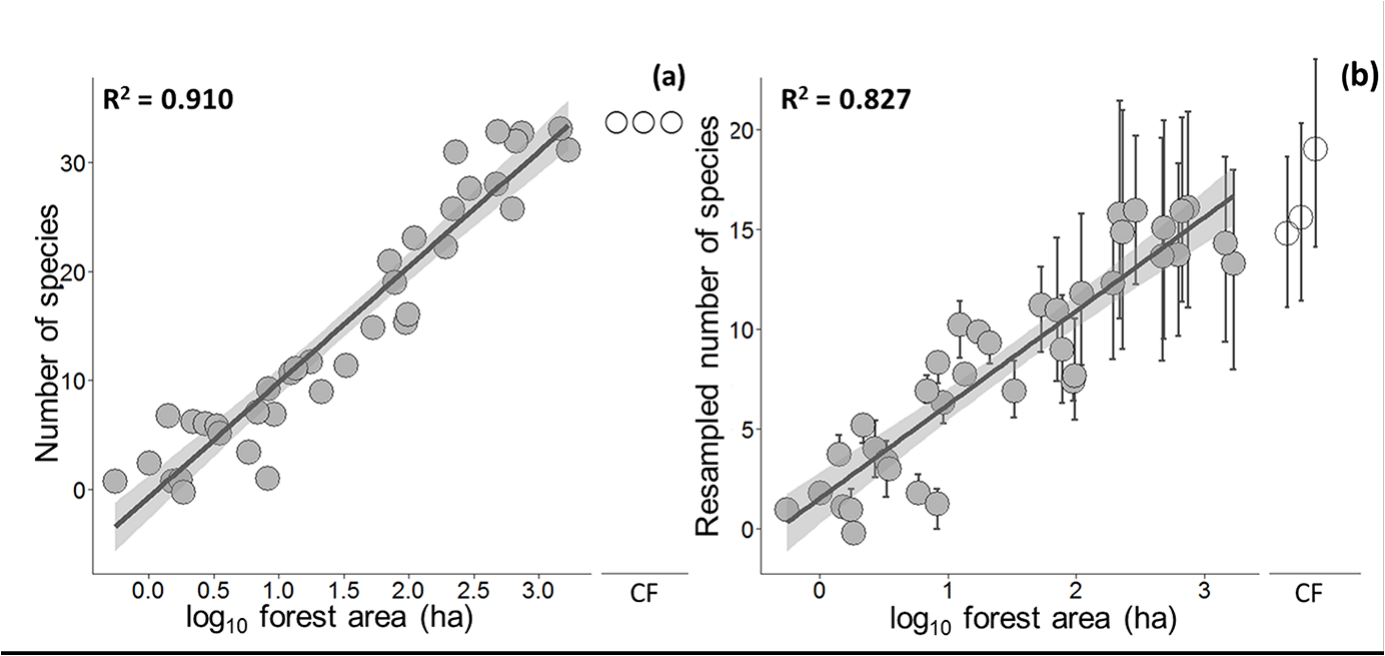
Relationships between forest patch area and species richness at 40 surveyed sites at Balbina considering (a) the total number of vertebrate species; and (b) the resampled mean (± SD) number of species per 500-m transect segment or individual camera trap (CT) station based on a standardized survey effort.

**Fig 2:**
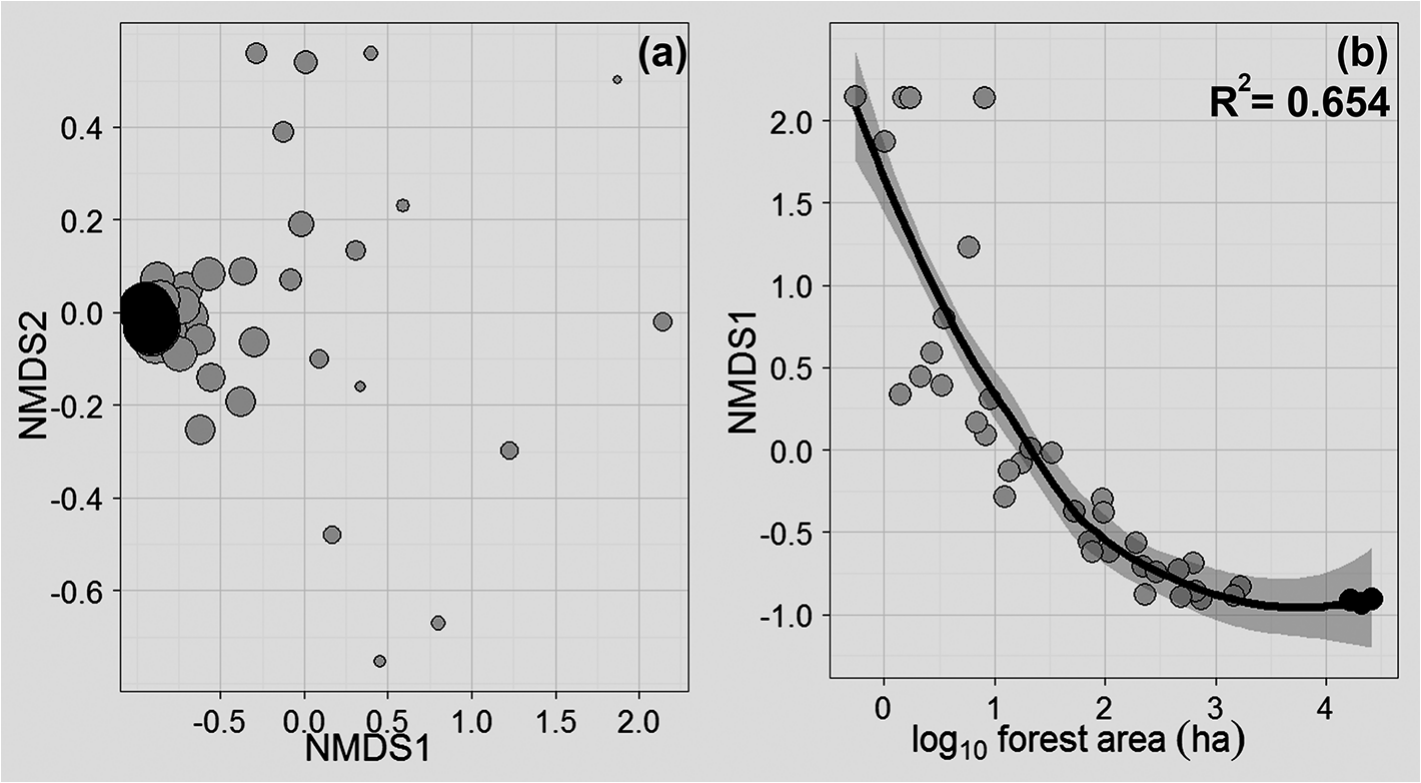
Nonmetric multidimensional scaling (NMDS) ordination plots based on the (a) Bray-Curtis similarity matrix of vertebrate species composition and (b) the relationship between the first NMDS axis and forest patch area. Circles are sized proportionally to (log_10_) forest patch area. Islands and continuous forest sites are shown in light and dark grey, respectively. Shaded area represents the 95% confidence region.

Balbina forest islands contained between 0 and 33 of all 35 vertebrate species recorded at least once (mean [SD] = 14.81 [11.18] species), whereas all three CFs contained 34 species. All species detected in CFs were also found in at least one island. Forest area alone explained 91% of the overall patch-scale variation in species richness considering a semi-log model and the combined occupancy data from all four sampling techniques (Fig 1A), and showed a steep SAR slope in the power model (*z-*value = 0.286; R^2^ = 0.886). Likewise, forest area explained 82.7% of the variation in survey-effort-standardized resampled species richness (semi-log model; Fig 1B). NMDS ordinations showed that vertebrate assemblage structure in large islands and CFs were more similar to one another than that in smaller islands, with islands <10 ha showing high levels of idiosyncratic dissimilarity depending on which small subset of species had been retained (Fig 2A). Overall, forest patch area was also an excellent predictor of species composition (R^2^_adj_ = 0.654, *p* <0.001), with islands >100 ha beginning to stabilize multivariate patterns of species similarity (Fig 2B). Forest area was also a powerful predictor of measures of aggregate vertebrate assemblage biomass (R^2^_adj_ = 0.769, *p* <0.001; S2A Fig) and functional diversity (R^2^_adj_ = 0.824, *p* <0.001; S2B Fig).

**Fig 3:**
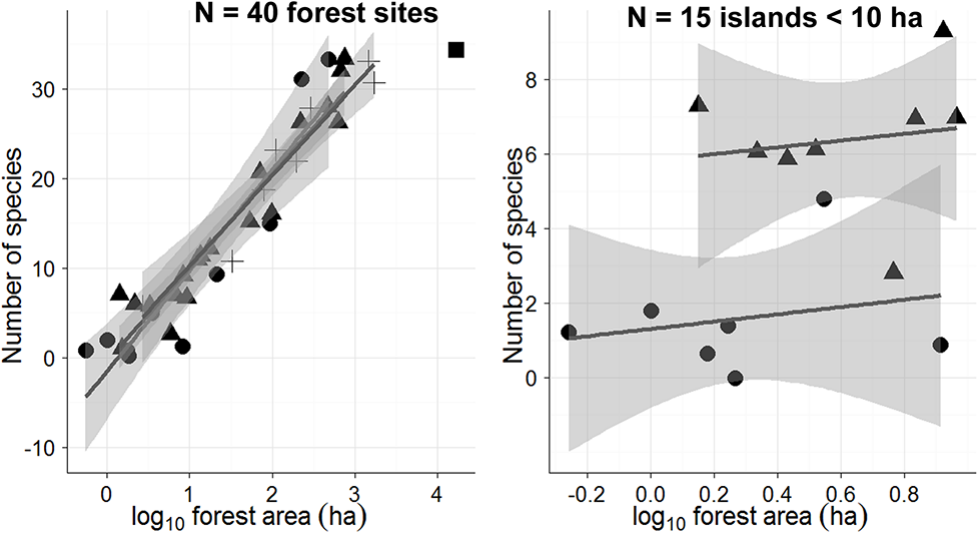
Relationships between forest patch area subjected to varying levels of burn severity and the total number of species persisting in (a) all 40 forest sites; and (b) only islands smaller than 10 ha. Symbols are coded according to burn severity (squares = unburnt; solid triangles = low burn severity; crosses = intermediate burn severity; solid circles = severely burnt).

Incorporating all seven explanatory variables, GLMs showed that forest patch area, ground fire severity and within-patch percentage of closed-forest canopy were significant predictors of species richness considering all 40 forest sites. However, patch area was the only significant predictor of species richness when we excluded CFs from the model (Table 1). In both cases, forest area captured a higher power of hierarchical partitioning, accounting for 64.8% of the relative importance among all significant variables considering all 40 forest sites. Excluding forest area from the analysis and considering only the 15 islands <10 ha, only fire severity was a significant predictor of species richness (Table 1). Levels of burn severity did not have a significant effect on the slopes of overall SARs for all sites (Fig 3A). However, the history of fire disturbance clearly modulated SARs in islands <10 ha driving many species to local extinction, whereby the seven severely burnt and eight unburnt small islands retained an average of 1.6 (range = 0–5) and 6.4 (3–9) species, respectively (Fig 3B).

**Table 1:** Summary of Generalized Linear Models (GLMs) of vertebrate species richness at (1) all 37 forest islands and three continuous forest sites; (2) the 37 islands only; and (3) 15 islands smaller than 10 ha across the BHR landscape.

Parameter	β	Unconditional SE	HP (%); IP
***N = 40***			
Intercept	0.100	0.452	
**area** (log_10_ x)	0.656	0.049	74.90; 0.648
**burn**	0.307	0.065	14.11; 0.003
**cc** **%**	0.010	0.003	8.39; 0.001
ba_*ff*_	-0.001	0.011	2.60
***N = 37***			
Intercept	1.211	0.286	
**area** (log_10_ x)	0.752	0.067	48.56
isolation	0.051	0.071	2.67
shape	-1.451	5.846	22.42
proximity	0.039	0.034	17.77
burn	0.000	0.074	3.04
cc%	0.001	0.003	3.44
ba_*ff*_	-0.003	0.014	2.10
***N = 15***			
Intercept	2.308	1.288	
isolation	0.037	0.225	1.45
shape	0.298	5.430	1.29
proximity	0.128	0.132	22.61
**burn**	-0.684	0.247	43.00
cc%	0.012	0.010	27.94
ba_*ff*_	-0.000	0.024	3.71

Coefficient estimates (β), their standard errors (SE), hierarchical partitioning (HP) of each variable, and independent power (IP) of each significant variable based on variance partition are shown. Significant variables are denoted in bold. See text for details on each variable.

### Predicting local extinctions across the entire landscape

**Fig 4:**
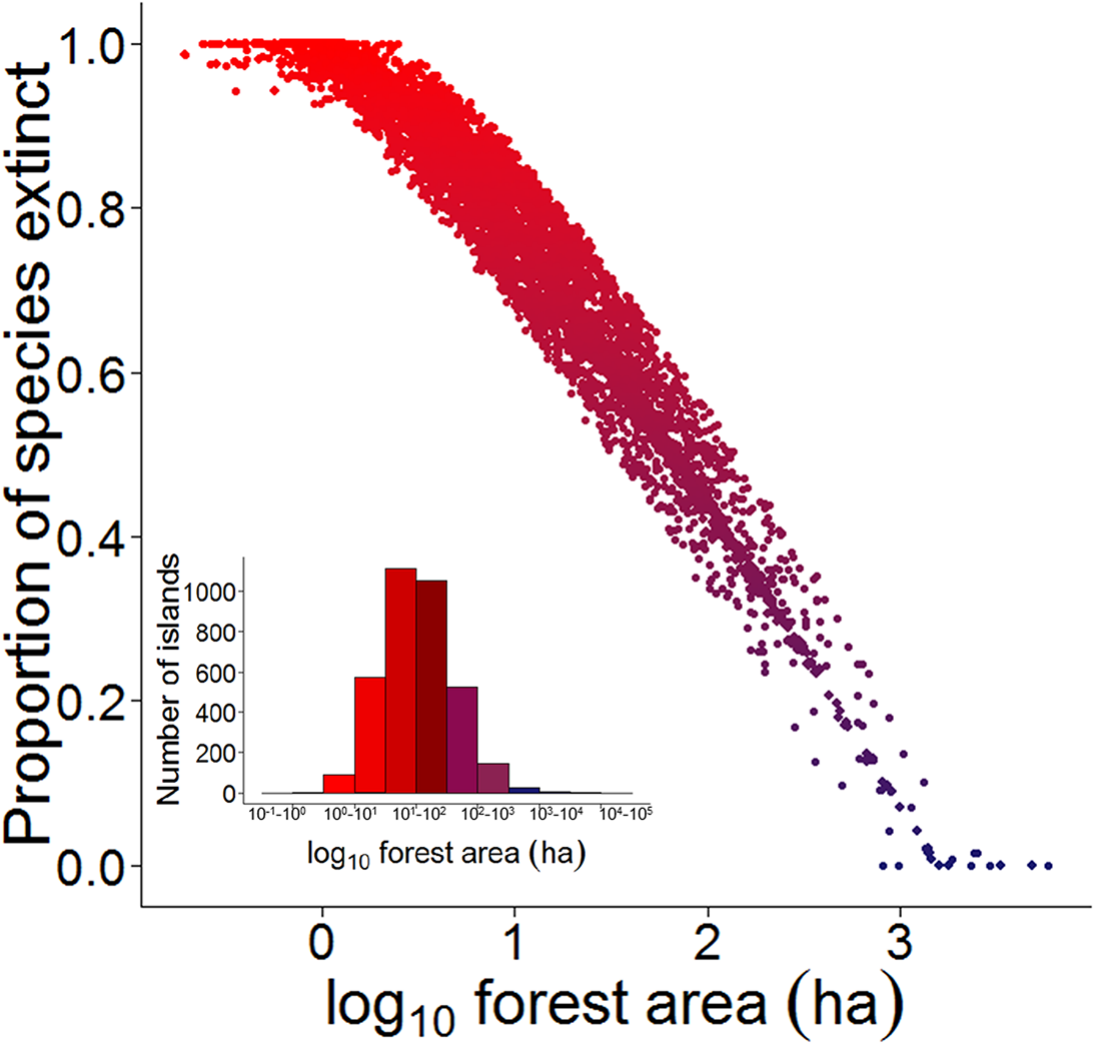
Proportion of forest vertebrate species predicted to have gone locally extinct as a function of forest patch area modelled for all 3,546 forest islands across the Balbina Hydroelectric Reservoir landscape. Heat color gradient in the scatterplot indicates the degree of local extinctions (increasing from blue to red) and matches the histogram describing the size distribution of all islands.

**Fig 5:**
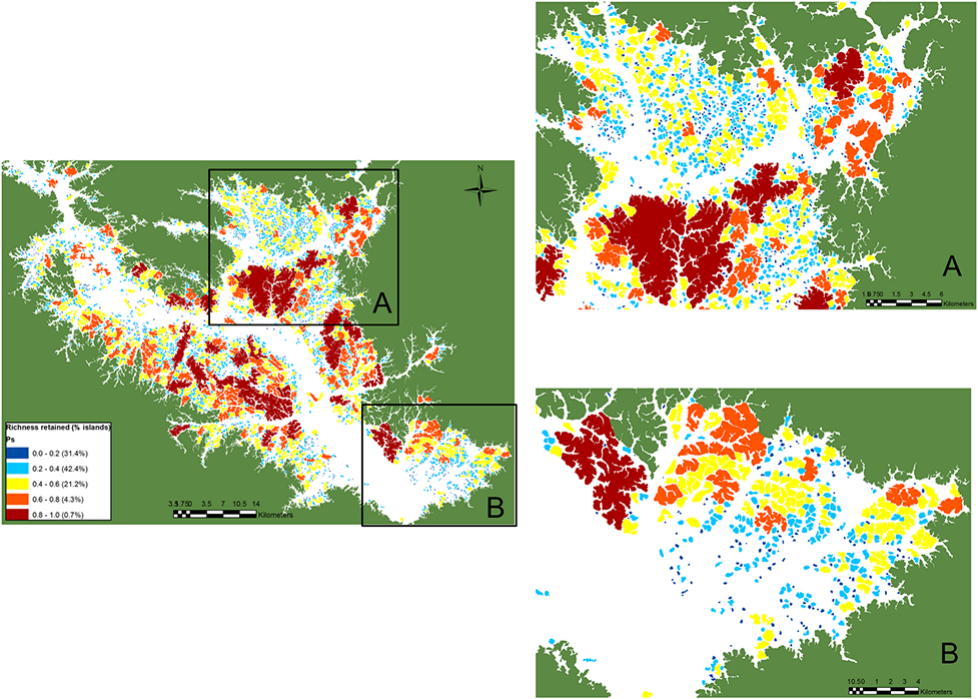
Heat map showing high intermediate and low priority sites for forest vertebrate conservation across the 3,546 islands within the Balbina Hydroelectric Reservoir landscape based on the empirical species-area relationship (R^2^ = 91%) derived from 37 surveyed islands. Islands are color-coded according to overall levels of species persistence (see legend).

We modelled patterns of vertebrate species richness and extinction across all 3,509 unsurveyed BHR islands using the observed semi-log linearized SAR model based on all 37 surveyed islands [S = c_s_ + z_s_ log(A)], where c_s_ = the intercept of the curve in arithmetic space and z_s_ = a direct measure of the initial and overall slope. Because forest area alone was a powerful predictor of species richness retained within islands (R^2^ = 91.0%), we used this SAR equation to predict the completeness of vertebrate assemblages for all 3,546 islands across the BHR. Our estimates predict that 95% of all islands retained fewer than 60% of the 35 vertebrate species considered here (Fig 4). All species surveyed are forest habitat generalists that occupy the wider habitat matrix of Amazonian *terra firme* forest landscapes [40]. Assuming that all landscape-wide species once occupied all sites within the archipelago prior to dam construction, we estimate an overall local extinction rate of 42.3% (548 of 1,295 populations) within the 37 islands surveyed. However, this rate increased to 70.3% (87,278 of 124,110 populations) when estimated for all 3,546 islands across the entire BHR landscape. Only islands larger than 475 ha still harbored a reasonably complete vertebrate community (≥80% of species), but this size threshold excludes all but 25 islands (0.7%) within the entire reservoir. We therefore identified the islands retaining the most complete vertebrate assemblages across the whole landscape (Fig 5).

## Discussion

Our study clearly demonstrates the colossal erosion in tropical forest vertebrate diversity induced by a major hydroelectric dam following a 26-year history of relaxation. We highlight the astounding long-term impact of the Balbina Dam on forest biodiversity within insular forest patches. Approximately 70% of all native medium to large vertebrate populations were predicted to have succumbed to local extinctions within the reservoir, and only 25 (0.7%) of all 3,546 islands currently retains four fifths of a full complement of species. These 25 islands comprise a total area of 34,481.3 ha, representing 29.2% of the combined area of all islands within the Balbina reservoir. Even though a species-rich vertebrate assemblage was retained in a few large islands, which is likely related to the strictly protected reserve established within the reservoir, the vast majority of islands failed to provide sufficiently large areas of high-quality habitat for the terrestrial/arboreal vertebrate fauna. This is mirrored in the aquatic realm, where the population of the largest apex carnivore in the reservoir—the giant otter—did not expand proportionally with the greatly augmented water-body at Balbina [41], suggesting that major hydroelectric reservoirs provide low-quality aquatic habitats and limited foodwebs to sustain top predators. Moreover, many vertebrate populations stranded in small forest islands are too small and far from safe thresholds of demographic or genetic viability, and will likely continue to pay an extinction debt [42–43]. We therefore surmise that the multifaceted terrestrial biodiversity impacts of mega hydroelectric dams have so far been severely neglected, emphasising the detrimental consequences on medium and large arboreal and terrestrial vertebrates.

At other major dammed tropical rivers worldwide, substantial declines in species diversity have been reported in the immediate aftermath of rising floodwaters and isolation [10,44], after a similar relaxation time as reported here [15, 45] and a much longer isolation history [12, 46]. In a hydroelectric reservoir in southern Thailand, small mammals virtually disappeared from a subset of 16 surveyed islands following 25 years of isolation, which was partly driven by an invasive rat species [15]. In another Amazonian dam (Tucuruí) with a similar age as Balbina, frog species richness also experienced a significant decline in islands, which was largely governed by island size [45]. In the BHR landscape, the topography inexorably contributed for the widespread of species losses observed here [24]. When a lowland tropical forest landscape is flooded, the mildly undulating topography favors the conversion of large areas of once unbroken forests into vast shallow lakes comprised of a large number of small islands. Given that management options for connecting forest islands and enhancing landscape-scale dispersal rates are unfeasible unless water levels drop substantially, insular biotas at BHR will likely experience even higher extinction rates in the long-term.

### Predictors of species loss

Several factors have been pinpointed to explain patterns of species extinction within tropical forest isolates. Under the island biogeography paradigm [47], area and isolation effects are consistently hailed as the prime predictors of species persistence in remaining habitat patches [48–49]. Other studies have emphasized the importance of considering the spatial arrangement of patches [50–51], the additive effects of anthropogenic disturbances such as hunting, logging and forest fires [28, 52–53], and habitat quality [54]. Additionally, matrix type has been recognized as a key driver of species loss, with true habitat islands showing higher declines in species richness than equivalent-sized remnants in terrestrial landscapes [12]. Using a multi-level approach, we considered a number of effects, in addition to area and isolation, to understand the main correlates of local extinction at the BHR forest islands.

Area was by far the most important predictor, explaining 91% and 82% of the overall variation in species richness and functional diversity, respectively, across all islands. In contrast, degree of isolation exerted no meaningful effect. Despite marked responses to landscape variables in large mammal studies elsewhere [55], we did not find a significant island proximity effect on species richness. However, fires that were likely initiated through anthropogenic sources of ignition, exerted a significant effect, but only in small islands (<10 ha). Hunting pressure is a strong predictor of medium and large mammal persistence across several fragmented tropical forest landscapes [21, 52, 56], and a key modulator of SARs in Neotropical primates [53]. However, our study islands were effectively protected from hunters by the Uatumã Biological Reserve. This is reflected in similar SAR slopes for different vertebrate size classes (S3 Fig), which is unlikely in a hunted landscape if hunters select large-bodied species. Indeed, the absence of subsistence and commercial hunting pressure has critically elevated the occupancy of game species, such as red brocket deer, collared peccary, and South American tapir, within the surveyed islands [24]. Forest habitat quality, expressed as the proportion of closed-canopy forest, exerted a significant effect when all forest sites were considered, but this variable was relatively unimportant compared to area effects. Finally, the inhospitable aquatic matrix appears to play a key role in explaining patterns of species persistence. The overall z-value of SARs at Balbina was considerably higher than those observed in analogous fragmentation ecology studies in Neotropical landscapes embedded within a terrestrial vegetation matrix [29, 52] and other taxonomic groups within true islands [12, 14]. Z-values can be considered as a strength metric of SARs, with steeper slopes consistently associated with low matrix permeability and immigration rates [57]. Our results thus reinforce the island occupancy role of the matrix, which is related to the low capacity of land vertebrates to traverse large open-water gaps in archipelagic landscapes (cf. [10]).

Lowland tropical forest islands created by major dams are therefore likely to succumb to higher rates of forest biodiversity loss than most forest remnants elsewhere, given that the predominantly small islands were isolated by a uniform non-habitat matrix. Fortunately, the Balbina archipelago has been effectively free from hunting ─ or else game vertebrate populations would fare even worse. Yet Balbina is the only Amazonian hydroelectric reservoir that is strictly protected by a Biological Reserve, suggesting that similar archipelagic landscapes are exposed to far worse negative synergisms between forest fragmentation and other anthropogenic disturbances. We therefore strongly recommend setting aside strictly protected forest reserves in future reservoirs as they can both mitigate extinction rates and ensure a stable experimental landscape setting for long-term ecological studies.

### Effects of forest fires

Vertebrate species composition was virtually identical across our three undisturbed continuous forest sites, but much less predictable in islands, particularly those <10 ha, which contained the most heterogeneous subsets of species, largely because of their recent fire disturbance history (Fig 3). Indeed, surface fires are an important driver of bird and mammal species loss in other neotropical fragmented forest landscapes [29, 58]. Islands subjected to a single event of severe fire perturbation experienced rapid rates of tree turnover, favoring fast-growing pioneers at the expense of old-growth tree species [17]. Indeed, insular tree assemblages were more susceptible to edge-related fire disturbance in small Balbina islands, given their greater perimeter-to-area ratios, which are aggravated by increased wind speed and greater desiccation [17]. As faunal assemblages are affected by compositional shifts in tree communities (e.g. [59]), extinction rates of several species in small islands were undoubtedly accelerated by both structural and compositional decay in tree assemblages. Fire perturbation is a key driver of population declines and/or local extinctions of several vertebrate species in Amazonian continuous forest settings [60]. Our results clearly show that small severely-burnt islands were most species-poor, highlighting the negative synergism between fire disturbance and area effects. Preventing surface fires within forest isolates would therefore reduce species losses in most true islands and habitat remnants.

### Policy Implications

Major hydroelectric dams are widely hailed as ‘green’ sources of renewable energy. However, the decision-making process on whether or not to build new major dams across lowland Amazonia and other tropical forest regions should be urgently reassessed. For those dams that are already built, protection against hunting, fire disturbance and unplanned settlements should be key mitigation measures to safeguard insular faunal communities. Apart from the poorly quantified social, economic and environmental costs of large dams—including displacements of local communities, loss in fishery revenues [3], wholesale shifts in aquatic faunal communities [41, 61], and significant greenhouse gas emissions [4]—we now provide clear evidence of widespread defaunation of forest islands even under a best-case protection scenario ensured by a large biological reserve. In addition to the 154 hydroelectric dams currently in operation across the Amazon, 277 new dams allocated to specific sites will likely be built over the next decades [62], with potential catastrophic effects on both aquatic and terrestrial biodiversity. Our study calls for decisive rethinking by policy-makers and energy strategists of future deployment of hydropower infrastructure in regions like Amazonia. Nevertheless, if a mega-dam is completely unavoidable, we suggest that habitat and biodiversity losses should be compensated by environmental offsets in the form of in situ or ex-situ protected areas. In addition to the entire reservoir area, adjacent continuous forest areas should also be protected within strictly forest reserves. The total extent of those protected areas should consider both the total inundation area (*i*.*e*. forests lost through submersion) and the summed areas of all islands below a given size threshold (<475 ha in the case of Balbina) which will likely lose at least four-fifths of their vertebrate diversity. These recommendations would meaningfully contribute to at least eight of the 20 Aichi Biodiversity Targets from the Convention on Biological Diversity ratified by several emergent developing countries, including Brazil (https://www.cbd.int/sp/targets/). Finally, we strongly encourage that erosion of populations of terrestrial and arboreal species in any landmass affected by dams, which so far have hardly entered the equation of total environmental costs, should be explicitly incorporated into environmental impact assessments of new dams.

## Supporting Information

S1 Fig Location of the Balbina Hydroelectric Reservoir (BHR) landscape in the State of Amazonas, Brazil, showing the 37 surveyed land-bridge islands (dark gray) and the three undisturbed continuous forest (CF) sites in the mainland (CF1, CF2 and CF3; shown in very dark gray). Black contours indicate 500-m buffer polygons around each island. All other 3,509 (unsurveyed) islands are shown in light gray.
(DOC)

S2 Fig Relationships between insular and continuous forest areas and a measure of aggregate vertebrate assemblage biomass (Figure A), and the vertebrate functional diversity persisting at 40 forest sites surveyed using four complementary sampling techniques across the Balbina Hydroelectric Reservoir landscape (Figure B). Shaded area represents 95% confidence region.
(DOC)

S3 Fig Species-area relationships as a function of body mass classes of forest vertebrate species (Small: ≤ 3kg; Medium: 3–9 kg; Large: ≥ 9 kg) surveyed within 37 islands and three continuous forest sites at the Balbina Hydroelectric Reservoir landscape. (DOC)

S1 Table Checklist of 35 vertebrate species surveyed within 40 forest sites at the Balbina archipelagic landscape and neighboring mainland areas and the sampling techniques quantifying the occupancy and abundance of each species. Solid circles (●) denote the most efficient survey technique for those species that could be detected by more than one method.
(DOC)
