# Supplementary material for: Widespread Forest Vertebrate Extinctions Induced by a Mega Hydroelectric Dam in Lowland Amazonia
Source: PLoS One. 2015 Jul 1;10(7):e0129818. doi: 10.1371/journal.pone.0129818 (PMC4488572; doi:10.1371/journal.pone.0129818)

**S1 Fig.** Location of the Balbina Hydroelectric Reservoir (BHR) landscape in the State of Amazonas, Brazil, showing the 37 surveyed land-bridge islands (dark gray) and the three undisturbed continuous forest (CF) sites in the mainland (CF1 CF2 and CF3; shown in very dark gray). Black contours indicate 500-m buffer polygons around each island. All other (unsurveyed) 3,509 islands are shown in light gray.


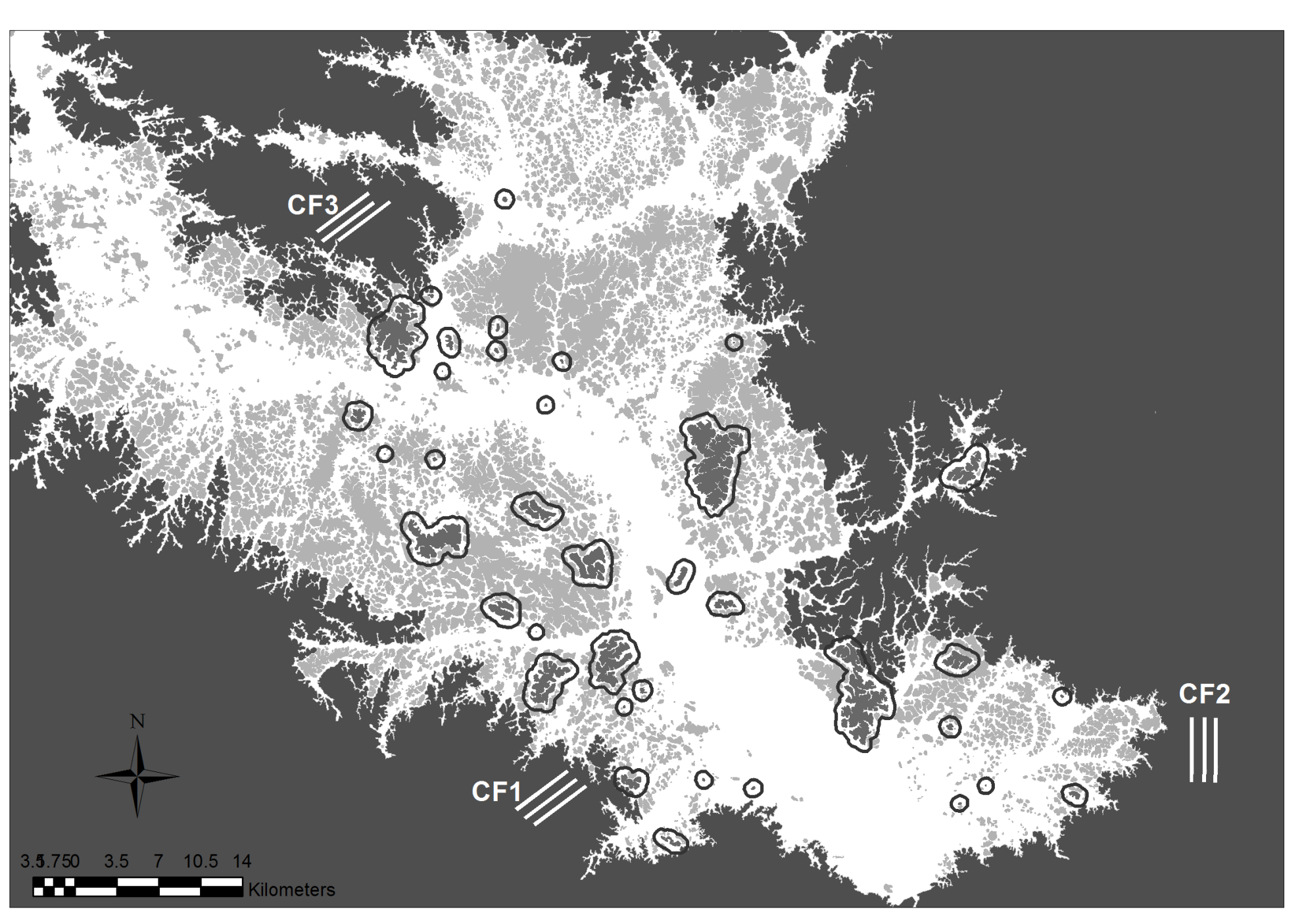

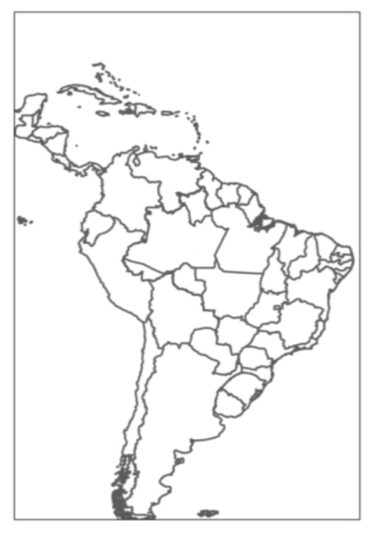

Supplement: S1 Fig — Black contours indicate 500-m buffer polygons around each island. All other 3,509 (unsurveyed) islands are shown in light gray. (DOC) [file pone.0129818.s001.doc]
