# Supplementary material for: Widespread Forest Vertebrate Extinctions Induced by a Mega Hydroelectric Dam in Lowland Amazonia
Source: PLoS One. 2015 Jul 1;10(7):e0129818. doi: 10.1371/journal.pone.0129818 (PMC4488572; doi:10.1371/journal.pone.0129818)

**S2 Fig.** Relationships between insular and continuous forest areas and a measure of aggregate vertebrate assemblage biomass (Figure A), and the vertebrate functional diversity persisting at 40 forest sites surveyed using four complementary sampling techniques across the Balbina Hydroelectric Reservoir landscape (Figure B). Shaded area represents 95% confidence region.


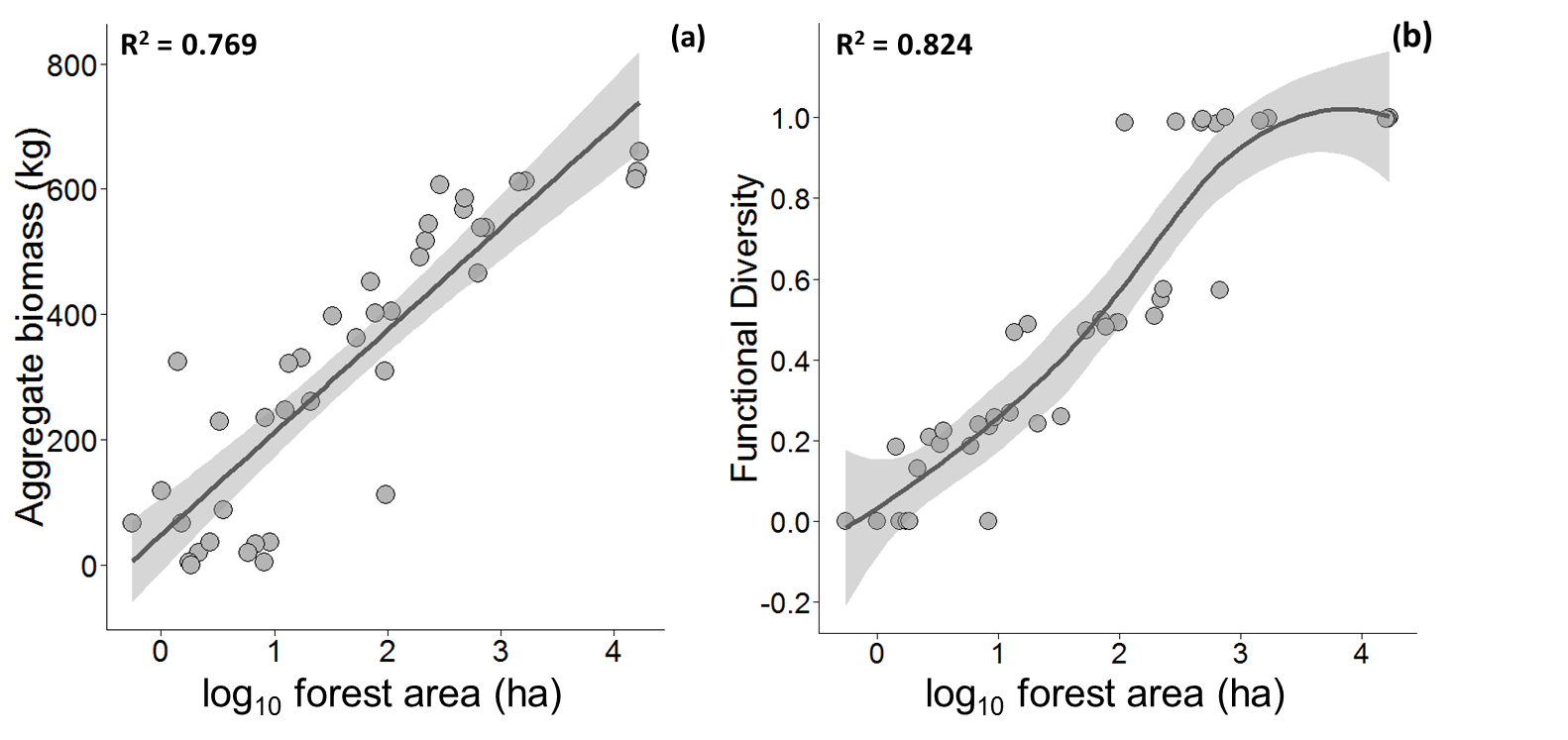

Supplement: S2 Fig — Shaded area represents 95% confidence region. (DOC) [file pone.0129818.s002.doc]
