# Supplementary material for: Widespread Forest Vertebrate Extinctions Induced by a Mega Hydroelectric Dam in Lowland Amazonia
Source: PLoS One. 2015 Jul 1;10(7):e0129818. doi: 10.1371/journal.pone.0129818 (PMC4488572; doi:10.1371/journal.pone.0129818)
Supplement: S3 Fig — (DOC) [file pone.0129818.s003.doc]

**S3 Fig.** Species-area relationships as a function of body mass classes of forest vertebrate species (Small: ≤ 3kg; Medium: 3-9 kg; Large: ≥ 9 kg) surveyed within 37 islands and three continuous forest sites at the Balbina Hydroelectric Reservoir landscape.

**
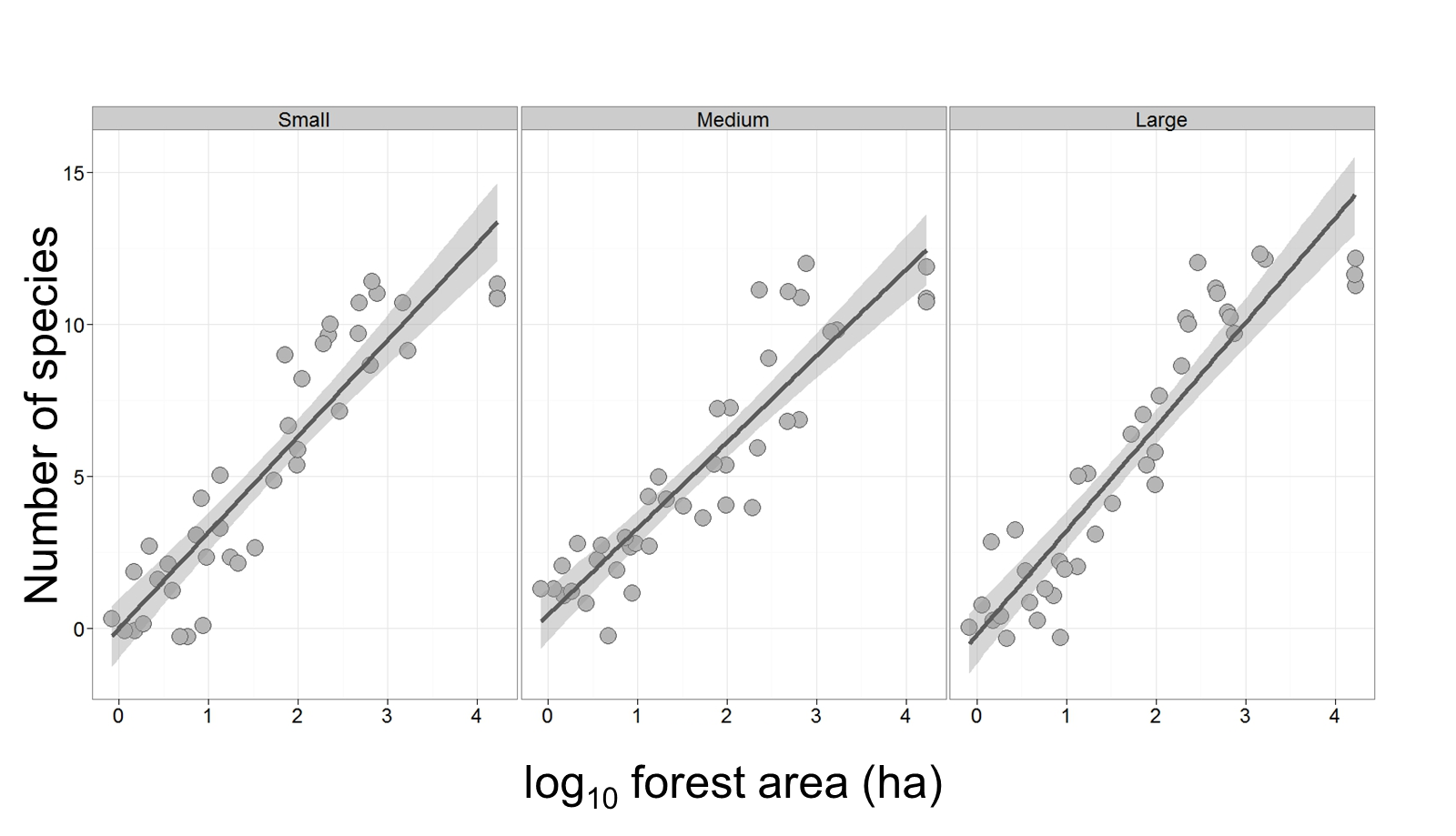
**
