# Supplementary material for: Widespread Forest Vertebrate Extinctions Induced by a Mega Hydroelectric Dam in Lowland Amazonia
Source: PLoS One. 2015 Jul 1;10(7):e0129818. doi: 10.1371/journal.pone.0129818 (PMC4488572; doi:10.1371/journal.pone.0129818)
Supplement: S1 Table — Solid circles (●) denote the most efficient survey technique for those species that could be detected by more than one method. (DOC) [file pone.0129818.s004.doc]

**S1 Table.** Checklist of 35 vertebrate species surveyed within 40 forest sites at the Balbina archipelagic landscape and neighbouring mainland areas and the sampling techniques quantifying the occupancy and abundance of each species. Solid circles (●) denote the most efficient survey technique for those species that could be detected by more than one method.

|  |  |  | **Survey method** | | | |
| --- | --- | --- | --- | --- | --- | --- |
| **Order/Family** | **Species** | **English vernacular name** | **Line-transect**  **censuses** | **Sign**  **surveys** | **Armadillo burrows**  **counts** | **Camera trapping** |
| **Mammals** |  |  |  |  |  |  |
| Artiodactyla/Cetartiodactyla | *Mazama americana* | Red brocket deer | X | ● |  | X |
| Artiodactyla/Cetartiodactyla | *Mazama nemorivaga* | Amazonian brown brocket deer | X | X |  | ● |
| Artiodacytla/Tayassuidae | *Pecari tajacu* | Collared peccary | X | X |  | ● |
| Artiodacytla/Tayassuidae | *Tayassu pecari* | White-lipped peccary | X | ● |  | X |
| Carnivora/Mustelidae | *Eira barbara* | Tayra | X |  |  | ● |
| Carnivora/Felidae | *Leopardus pardalis* | Ocelot | X | X |  | ● |
| Carnivora/ Felidae | *Leopardus wiedii* | Margay |  |  |  | ● |
| Carnivora/ Felidae | *Panthera onca* | Jaguar | X | X |  | ● |
| Carnivora/ Felidae | *Puma concolor* | Puma | X | X |  | ● |
| Carnivora/ Felidae | *Puma yaguaroundi* | Jaguarundi | X |  |  | ● |
| Carnivora/Procyonidae | *Nasua nasua* | South American coati | X | X |  | ● |
| Cingulata/Dasypodidae | *Cabassous unicinctus* | Southern naked-tailed armadillo | X | X | ● |  |
| Cingulata/Dasypodidae | *Dasypus kappleri* | Greater long-nosed armadillo |  | X | ● | X |
| Cingulata/Dasypodidae | *Dasypus novemcinctus* | Nine-banded armadillo |  | X | X | ● |
| Cingulata/Dasypodidae | *Priodontes maximus* | Giant armadillo |  | X | X | ● |
| Perissodactyla/Tapiridae | *Tapirus terrestris* | South American tapir | X | ● |  | X |
| Pilosa/Myrmecophagidae | *Myrmecophaga tridactyla* | Giant anteater | X | X |  | ● |
| Pilosa/Myrmecophagidae | *Tamandua tetradactyla* | Southern tamandua | ● |  |  | X |
| Primates/Atelidae | *Alouatta macconelli* | Red howler monkey | ● | X |  |  |
| Primates/Atelidae | *Ateles paniscus* | Black spider monkey | ● | X |  |  |
| Primates/Pitheciidae | *Chiropotes sagulatus* | Northern bearded saki | X |  |  |  |
| Primates/Pitheciidae | *Pithecia chrysocephala* | Golden-faced saki | X |  |  |  |
| Primates/Callithrichidae | *Saguinus midas* | Golden-handed tamarin | X |  |  |  |
| Primates/Cebidae | *Saimiri sciureus* | Squirrel monkey | X |  |  |  |
| Primates/Cebidae | *Sapajus apella* | Brown capuchin monkey | X |  |  |  |
| Rodentia/Cuniculidae | *Cuniculus paca* | Lowland paca |  | X |  | ● |
| Rodentia/Dasyproctidae | *Dasyprocta leporina* | Red-rumped agouti | X | X |  | ● |
| Rodentia/Dasyproctidae | *Myoprocta acouchy* | Red acouchi | ● | X |  | X |
| Rodentia/Sciuridae | *Guerlinguetus aestuans* | Brazilian squirrel | ● | X |  | X |
| **Birds** |  |  |  |  |  |  |
| Galliformes/Cracidae | *Penelope marail* | Marail guan | ● |  |  |  |
| Galliformes/Cracidae | *Crax alector* | Black curassow | X |  |  | ● |
| Gruiformes/Psophiidae | *Psophia crepitans* | Grey-winged trumpeter | X |  |  | ● |
| Tinamiformes/Tinamidae | *Tinamus major* | Great tinamou | ● | X |  | X |
| **Reptiles** |  |  |  |  |  |  |
| Testudines/Testudinidae | *Chelonoidis carbonaria* | Red-footed tortoise | ● |  |  |  |
| Testudines/Testudinidae | *Chelonoidis denticulata* | Yellow-footed tortoise | ● |  |  |  |
